# (*S*)-(+)-1-(1-Naphth­yl)-1-(2-thienylmethyl­ene)ethyl­amine

**DOI:** 10.1107/S1600536809022375

**Published:** 2009-06-20

**Authors:** Armando Espinosa Leija, Guadalupe Hernández, Roberto Portillo, René Gutiérrez, Sylvain Bernès

**Affiliations:** a Facultad de Ciencias Químicas, UANL, Licenciatura en Química Industrial, Ciudad Universitaria, Monterrey, NL, Mexico; b Laboratorio de Síntesis de Complejos, Facultad de Ciencias Químicas, BUAP, AP 1067, 72001 Puebla, Pue., Mexico; c DEP Facultad de Ciencias Químicas, UANL, Guerrero y Progreso S/N, Col. Treviño, 64570 Monterrey, NL, Mexico

## Abstract

The title chiral imine, C_17_H_15_NS, has been obtained *via* a direct synthesis route. The imine group displays the common *E* configuration, and is almost coplanar with the thio­phene heterocycle; the dihedral angle between the C=N—C group and the thio­phene ring is 5.1 (8)°. In contrast, the naphthyl group makes an angle of 83.79 (13)° with the thio­phene ring. The observed solid-state mol­ecular conformation is suitable for the use of this mol­ecule as an *N*,*S*-bidentate Schiff base ligand. The mol­ecular packing features double C—H⋯π inter­actions between naphthyl groups of neighboring mol­ecules, which form chains in the [100] direction. The crystal structure is further stabilized by a short C—H⋯π contact involving the methyl group and one ring of a naphthyl group. The resulting two-dimensional network is completed by a weak inter­molecular C—H(imine)⋯π(thio­phene) inter­action.

## Related literature

For background to direct synthesis, see: Tanaka & Toda (2000 ▶); Jeon *et al.* (2005 ▶); Tovar *et al.* (2007 ▶). For the configuration and conformation of imines derived from thio­phene, see: Arjona *et al.* (1986 ▶).
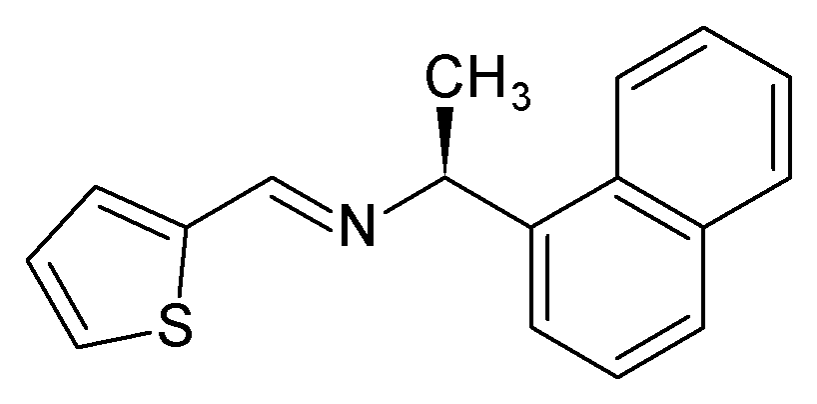


## Experimental

### 

#### Crystal data

C_17_H_15_NS
*M* *_r_* = 265.36
Orthorhombic, 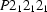

*a* = 5.5274 (14) Å
*b* = 7.990 (2) Å
*c* = 31.517 (8) Å
*V* = 1392.0 (6) Å^3^
*Z* = 4
Mo *K*α radiation
μ = 0.22 mm^−1^
*T* = 298 K
0.50 × 0.36 × 0.04 mm

#### Data collection

Siemens P4 diffractometer
Absorption correction: ψ scan (*XSCANS*; Siemens, 1996 ▶) *T*_min_ = 0.802, *T*_max_ = 0.991
4462 measured reflections
2446 independent reflections
1280 reflections with *I* > 2σ(*I*)
*R*_int_ = 0.045
2 standard reflections every 48 reflections intensity decay: 1.8%

#### Refinement

*R*[*F* ^2^ > 2σ(*F* ^2^)] = 0.059
*wR*(*F* ^2^) = 0.174
*S* = 1.56
2446 reflections
174 parameters
H-atom parameters constrained
Δρ_max_ = 0.31 e Å^−3^
Δρ_min_ = −0.39 e Å^−3^
Absolute structure: Flack (1983 ▶), 946 Friedel pairs
Flack parameter: 0.2 (2)

### 

Data collection: *XSCANS* (Siemens, 1996 ▶); cell refinement: *XSCANS*; data reduction: *XSCANS*; program(s) used to solve structure: *SHELXS97* (Sheldrick, 2008 ▶); program(s) used to refine structure: *SHELXL97* (Sheldrick, 2008 ▶); molecular graphics: *Mercury* (Macrae *et al.*, 2006 ▶); software used to prepare material for publication: *SHELXL97*.

## Supplementary Material

Crystal structure: contains datablocks I, global. DOI: 10.1107/S1600536809022375/wn2331sup1.cif

Structure factors: contains datablocks I. DOI: 10.1107/S1600536809022375/wn2331Isup2.hkl

Additional supplementary materials:  crystallographic information; 3D view; checkCIF report

## Figures and Tables

**Table 1:** Hydrogen-bond geometry (Å, °)

*D*—H⋯*A*	*D*—H	H⋯*A*	*D*⋯*A*	*D*—H⋯*A*
C9—H9*B*⋯CgA^i^	0.96	2.85	3.682 (6)	145
C6—H6*A*⋯CgB^ii^	0.93	3.03	3.891 (5)	155
C13—H13*A*⋯CgC^iii^	0.93	3.54	4.399 (6)	155
C15—H15*A*⋯CgA^iii^	0.93	3.22	4.030 (6)	147

Symmetry codes: (i) 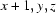; (ii) 
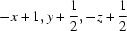
; (iii) 
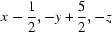
. *CgA* is the centroid of ring C10–C14/C19, *CgB* is the centroid of the thio­phene ring and *CgC* is the centroid of ring C14–C19.

**Fig. 1.**
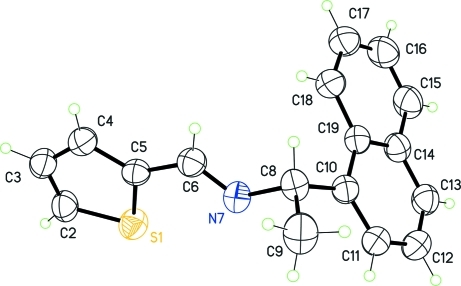
The title molecule with displacement ellipsoids for non-H atoms shown at the 30% probability level. Hydrogen atoms are shown as spheres of arbitrary radius.

**Fig. 2.**
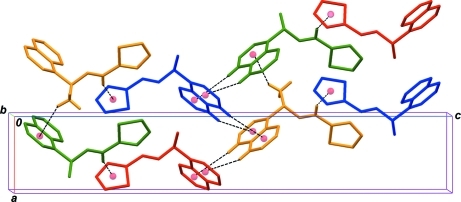
A part of the crystal structure of the title compound, viewed down [010]. The color scheme is used for the sake of clarity. Dashed lines represent C—H···π interactions in the crystal structure, and the centroids of involved π systems have been represented by red spheres. H atoms not involved in the network of intermolecular contacts have been omitted.

## supplementary crystallographic information

### Comment

Nowadays, there is an increasing interest in the use of environmentally benign reagents and conditions, leading particularly to solvent-free procedures. Avoiding organic solvents during the reactions in organic synthesis affords clean, efficient and economical features: safety is largely increased, working is considerably simplified, cost is reduced, increased amounts of reactants can be used, *etc*. (Tanaka & Toda, 2000; Jeon *et al.*, 2005).

On the other hand, imines continue to attract much attention, mainly due to their versatile coordination behavior and the interesting properties of their metal complexes. Continuing our work on the synthesis of chiral imines (Tovar *et al.*, 2007), we synthesized the title compound under solvent-free conditions (see *Experimental*) and report here its X-ray crystal structure.

The molecule is stabilized in the solid state as an *E*-*trans* aldimine (Fig. 1), which has been shown to be the preferred configuration for imine systems derived from thiophene (Arjona *et al.*, 1986). By conjugation, the imine group C6/N7/C8 is almost coplanar with the thiophene ring S1/C2/C3/C4/C5, with a dihedral angle of 5.1 (8)°. In contrast, the naphthyl group is almost normal to the thiophene ring, at 83.79 (13)°. The crystal packing features a number of intermolecular C—H···π contacts (Fig. 2), the strongest involving the methyl group and a naphthyl group of a symmetry-related molecule. Naphthyl systems aggregate through double C—H···π interactions, forming chains along the [100] direction. The set of contacts results in a two-dimensional framework of efficiently stacked molecules.

### Experimental

Under solvent-free conditions, (*S*)-(-)-(1-naphthyl)ethylamine (213 mg, 1.24 mmol) and 2-thiophenecarboxaldehyde (139 mg, 1.24 mmol) were mixed at 298 K, giving a white solid. The crude product was recrystallized from CH_2_Cl_2_, affording colorless crystals of the title compound. Yield 87%; m.p. 345 K. Analytical data are in agreement with the structure determined by X-ray diffraction (see archived CIF).

### Refinement

The title molecule crystallizes as thin plates, and the selected crystal was a poorly diffracting sample, limiting data resolution. All H atoms were placed in idealized positions and refined as riding on their carrier C atoms, with bond lengths fixed to 0.93 (aromatic CH), 0.96 (methyl CH_3_), and 0.98 Å (methine CH). Isotropic displacement parameters were calculated as *U*_iso_(H) = 1.5*U*_eq_(carrier atom) for the methyl group and *U*_iso_(H) = 1.2*U*_eq_(carrier atom) otherwise. The absolute configuration was assigned by refinement of a Flack parameter, and agrees with the chirality expected from the synthetic route.

### Crystal data

**Table:**

C_17_H_15_NS	*D*_x_ = 1.266 Mg m^−^^3^
*M**_r_* = 265.36	Melting point: 345 K
Orthorhombic, *P*2_1_2_1_2_1_	Mo *K*α radiation, λ = 0.71073 Å
Hall symbol: P 2ac 2ab	Cell parameters from 68 reflections
*a* = 5.5274 (14) Å	θ = 4.9–11.5°
*b* = 7.990 (2) Å	µ = 0.22 mm^−^^1^
*c* = 31.517 (8) Å	*T* = 298 K
*V* = 1392.0 (6) Å^3^	Plate, colourless
*Z* = 4	0.50 × 0.36 × 0.04 mm
*F*(000) = 560	

### Data collection

**Table:**

Siemens P4 diffractometer	1280 reflections with *I* > 2σ(*I*)
Radiation source: fine-focus sealed tube	*R*_int_ = 0.045
graphite	θ_max_ = 25.0°, θ_min_ = 2.6°
ω scans	*h* = −6→6
Absorption correction: ψ scan (*XSCANS*; Siemens, 1996)	*k* = −9→9
*T*_min_ = 0.802, *T*_max_ = 0.991	*l* = −37→37
4462 measured reflections	2 standard reflections every 48 reflections
2446 independent reflections	intensity decay: 1.8%

### Refinement

**Table:**

Refinement on *F*^2^	Hydrogen site location: inferred from neighbouring sites
Least-squares matrix: full	H-atom parameters constrained
*R*[*F*^2^ > 2σ(*F*^2^)] = 0.059	*w* = 1/[σ^2^(*F*_o_^2^) + (0.05*P*)^2^] where *P* = (*F*_o_^2^ + 2*F*_c_^2^)/3
*wR*(*F*^2^) = 0.174	(Δ/σ)_max_ < 0.001
*S* = 1.56	Δρ_max_ = 0.31 e Å^−^^3^
2446 reflections	Δρ_min_ = −0.39 e Å^−^^3^
174 parameters	Extinction correction: *SHELXL97* (Sheldrick, 2008), Fc^*^=kFc[1+0.001xFc^2^λ^3^/sin(2θ)]^-1/4^
0 restraints	Extinction coefficient: 0.046 (6)
0 constraints	Absolute structure: Flack (1983), 946 Friedel pairs
Primary atom site location: structure-invariant direct methods	Flack parameter: 0.2 (2)
Secondary atom site location: difference Fourier map	

### Fractional atomic coordinates and isotropic or equivalent isotropic displacement parameters (Å^2^)

**Table:**

	*x*	*y*	*z*	*U*_iso_*/*U*_eq_	
S1	0.0890 (3)	0.77472 (18)	0.23563 (4)	0.0851 (5)	
C2	0.0387 (11)	0.7930 (7)	0.28848 (16)	0.0838 (16)	
H2A	−0.0852	0.7377	0.3027	0.101*	
C3	0.1958 (10)	0.8951 (7)	0.30739 (17)	0.0808 (16)	
H3A	0.1920	0.9189	0.3363	0.097*	
C4	0.3675 (11)	0.9631 (6)	0.27948 (16)	0.0765 (15)	
H4A	0.4906	1.0359	0.2875	0.092*	
C5	0.3292 (8)	0.9077 (6)	0.23880 (16)	0.0637 (12)	
C6	0.4666 (10)	0.9518 (6)	0.20103 (16)	0.0709 (14)	
H6A	0.5865	1.0331	0.2030	0.085*	
N7	0.4278 (8)	0.8830 (5)	0.16565 (12)	0.0732 (11)	
C8	0.5830 (11)	0.9318 (6)	0.12949 (14)	0.0743 (14)	
H8A	0.6648	1.0374	0.1362	0.089*	
C9	0.7706 (10)	0.7968 (8)	0.12312 (17)	0.0982 (19)	
H9A	0.8835	0.7989	0.1462	0.147*	
H9B	0.8550	0.8161	0.0970	0.147*	
H9C	0.6924	0.6896	0.1221	0.147*	
C10	0.4174 (10)	0.9587 (6)	0.09162 (14)	0.0679 (13)	
C11	0.3745 (10)	0.8354 (6)	0.06322 (15)	0.0771 (14)	
H11A	0.4564	0.7344	0.0662	0.093*	
C12	0.2109 (10)	0.8542 (7)	0.02938 (16)	0.0837 (17)	
H12A	0.1858	0.7662	0.0106	0.100*	
C13	0.0910 (12)	0.9986 (7)	0.02414 (17)	0.0835 (16)	
H13A	−0.0180	1.0099	0.0019	0.100*	
C14	0.1289 (10)	1.1329 (7)	0.05212 (16)	0.0757 (14)	
C15	0.0159 (12)	1.2883 (8)	0.04640 (19)	0.101 (2)	
H15A	−0.0913	1.3014	0.0239	0.121*	
C16	0.0570 (16)	1.4195 (9)	0.0723 (2)	0.110 (2)	
H16A	−0.0224	1.5205	0.0677	0.132*	
C17	0.2162 (13)	1.4043 (8)	0.1058 (2)	0.0980 (19)	
H17A	0.2446	1.4950	0.1236	0.118*	
C18	0.3321 (11)	1.2564 (7)	0.11269 (16)	0.0850 (16)	
H18A	0.4378	1.2477	0.1355	0.102*	
C19	0.2965 (10)	1.1158 (6)	0.08620 (14)	0.0702 (14)	

### Atomic displacement parameters (Å^2^)

**Table:**

	*U*^11^	*U*^22^	*U*^33^	*U*^12^	*U*^13^	*U*^23^
S1	0.0830 (9)	0.0941 (10)	0.0780 (9)	−0.0071 (9)	−0.0027 (8)	−0.0051 (8)
C2	0.092 (4)	0.093 (4)	0.066 (3)	0.000 (4)	0.010 (3)	0.006 (3)
C3	0.093 (4)	0.083 (4)	0.067 (3)	0.008 (4)	−0.008 (3)	−0.003 (3)
C4	0.085 (4)	0.074 (3)	0.071 (3)	−0.009 (3)	−0.001 (3)	−0.005 (3)
C5	0.058 (3)	0.063 (3)	0.070 (3)	−0.006 (2)	−0.003 (2)	−0.002 (3)
C6	0.069 (4)	0.071 (3)	0.073 (3)	−0.002 (3)	−0.005 (3)	0.000 (3)
N7	0.075 (3)	0.082 (3)	0.062 (2)	0.001 (3)	0.003 (2)	0.001 (2)
C8	0.078 (3)	0.079 (3)	0.066 (3)	0.000 (3)	0.003 (3)	0.008 (3)
C9	0.086 (4)	0.119 (5)	0.090 (4)	0.029 (4)	0.004 (3)	0.007 (4)
C10	0.073 (3)	0.069 (3)	0.062 (3)	0.002 (3)	0.001 (3)	0.003 (3)
C11	0.089 (4)	0.073 (3)	0.070 (3)	0.004 (3)	0.001 (3)	−0.001 (3)
C12	0.091 (4)	0.085 (4)	0.076 (4)	−0.002 (4)	−0.003 (3)	−0.010 (3)
C13	0.088 (4)	0.093 (4)	0.070 (3)	−0.002 (4)	−0.007 (3)	0.003 (3)
C14	0.077 (4)	0.078 (3)	0.072 (3)	0.006 (3)	0.002 (3)	0.008 (3)
C15	0.117 (5)	0.096 (4)	0.090 (4)	0.026 (4)	−0.006 (4)	0.018 (4)
C16	0.134 (6)	0.083 (4)	0.112 (5)	0.024 (5)	0.014 (5)	0.013 (4)
C17	0.117 (5)	0.078 (4)	0.099 (4)	0.007 (4)	0.017 (4)	−0.005 (4)
C18	0.097 (4)	0.077 (4)	0.081 (3)	−0.006 (4)	0.009 (3)	−0.007 (3)
C19	0.077 (3)	0.072 (3)	0.062 (3)	−0.001 (3)	0.006 (3)	0.003 (3)

### Geometric parameters (Å, °)

**Table:**

S1—C2	1.695 (5)	C10—C11	1.352 (6)
S1—C5	1.703 (5)	C10—C19	1.432 (6)
C2—C3	1.332 (7)	C11—C12	1.407 (7)
C2—H2A	0.9300	C11—H11A	0.9300
C3—C4	1.404 (7)	C12—C13	1.341 (7)
C3—H3A	0.9300	C12—H12A	0.9300
C4—C5	1.373 (6)	C13—C14	1.405 (7)
C4—H4A	0.9300	C13—H13A	0.9300
C5—C6	1.455 (6)	C14—C15	1.401 (7)
C6—N7	1.261 (5)	C14—C19	1.425 (7)
C6—H6A	0.9300	C15—C16	1.349 (7)
N7—C8	1.479 (6)	C15—H15A	0.9300
C8—C9	1.509 (7)	C16—C17	1.378 (9)
C8—C10	1.519 (7)	C16—H16A	0.9300
C8—H8A	0.9800	C17—C18	1.361 (8)
C9—H9A	0.9600	C17—H17A	0.9300
C9—H9B	0.9600	C18—C19	1.414 (6)
C9—H9C	0.9600	C18—H18A	0.9300
			
C2—S1—C5	91.0 (3)	C11—C10—C8	121.5 (5)
C3—C2—S1	112.7 (5)	C19—C10—C8	119.9 (4)
C3—C2—H2A	123.7	C10—C11—C12	122.5 (5)
S1—C2—H2A	123.7	C10—C11—H11A	118.8
C2—C3—C4	113.4 (5)	C12—C11—H11A	118.8
C2—C3—H3A	123.3	C13—C12—C11	120.2 (5)
C4—C3—H3A	123.3	C13—C12—H12A	119.9
C5—C4—C3	110.8 (5)	C11—C12—H12A	119.9
C5—C4—H4A	124.6	C12—C13—C14	120.4 (6)
C3—C4—H4A	124.6	C12—C13—H13A	119.8
C4—C5—C6	127.2 (4)	C14—C13—H13A	119.8
C4—C5—S1	112.1 (4)	C15—C14—C13	122.0 (5)
C6—C5—S1	120.6 (4)	C15—C14—C19	118.1 (5)
N7—C6—C5	121.9 (5)	C13—C14—C19	119.8 (5)
N7—C6—H6A	119.0	C16—C15—C14	122.4 (6)
C5—C6—H6A	119.0	C16—C15—H15A	118.8
C6—N7—C8	117.9 (4)	C14—C15—H15A	118.8
N7—C8—C9	108.2 (4)	C15—C16—C17	120.1 (6)
N7—C8—C10	107.0 (4)	C15—C16—H16A	119.9
C9—C8—C10	114.2 (4)	C17—C16—H16A	119.9
N7—C8—H8A	109.1	C18—C17—C16	119.9 (6)
C9—C8—H8A	109.1	C18—C17—H17A	120.0
C10—C8—H8A	109.1	C16—C17—H17A	120.0
C8—C9—H9A	109.5	C17—C18—C19	122.0 (6)
C8—C9—H9B	109.5	C17—C18—H18A	119.0
H9A—C9—H9B	109.5	C19—C18—H18A	119.0
C8—C9—H9C	109.5	C18—C19—C14	117.3 (5)
H9A—C9—H9C	109.5	C18—C19—C10	124.1 (5)
H9B—C9—H9C	109.5	C14—C19—C10	118.5 (5)
C11—C10—C19	118.5 (5)		
			
C5—S1—C2—C3	0.2 (4)	C11—C12—C13—C14	0.6 (9)
S1—C2—C3—C4	−0.4 (6)	C12—C13—C14—C15	176.8 (5)
C2—C3—C4—C5	0.5 (7)	C12—C13—C14—C19	0.2 (9)
C3—C4—C5—C6	179.1 (5)	C13—C14—C15—C16	−177.9 (6)
C3—C4—C5—S1	−0.4 (6)	C19—C14—C15—C16	−1.3 (9)
C2—S1—C5—C4	0.1 (4)	C14—C15—C16—C17	0.5 (11)
C2—S1—C5—C6	−179.4 (4)	C15—C16—C17—C18	−0.1 (10)
C4—C5—C6—N7	174.2 (5)	C16—C17—C18—C19	0.6 (9)
S1—C5—C6—N7	−6.4 (6)	C17—C18—C19—C14	−1.4 (8)
C5—C6—N7—C8	−177.8 (4)	C17—C18—C19—C10	178.7 (5)
C6—N7—C8—C9	101.4 (5)	C15—C14—C19—C18	1.7 (7)
C6—N7—C8—C10	−135.0 (5)	C13—C14—C19—C18	178.4 (5)
N7—C8—C10—C11	−94.5 (6)	C15—C14—C19—C10	−178.5 (5)
C9—C8—C10—C11	25.3 (7)	C13—C14—C19—C10	−1.7 (7)
N7—C8—C10—C19	83.5 (6)	C11—C10—C19—C18	−177.6 (5)
C9—C8—C10—C19	−156.7 (5)	C8—C10—C19—C18	4.3 (8)
C19—C10—C11—C12	−1.9 (8)	C11—C10—C19—C14	2.5 (7)
C8—C10—C11—C12	176.1 (5)	C8—C10—C19—C14	−175.5 (5)
C10—C11—C12—C13	0.3 (8)		

### Hydrogen-bond geometry (Å, °)

**Table:**

*D*—H···*A*	*D*—H	H···*A*	*D*···*A*	*D*—H···*A*
C9—H9B···CgA^i^	0.96	2.85	3.682 (6)	145
C6—H6A···CgB^ii^	0.93	3.03	3.891 (5)	155
C13—H13A···CgC^iii^	0.93	3.54	4.399 (6)	155
C15—H15A···CgA^iii^	0.93	3.22	4.030 (6)	147

Symmetry codes: (i) *x*+1, *y*, *z*; (ii) −*x*+1, *y*+1/2, −*z*+1/2; (iii) *x*−1/2, −*y*+5/2, −*z*.
